# Atypical Pathogenicity of Avian Influenza (H3N1) Virus Involved in Outbreak, Belgium, 2019

**DOI:** 10.3201/eid2608.191338

**Published:** 2020-08

**Authors:** Mieke Steensels, Philippe Gelaude, Steven Van Borm, Thierry Van Den Berg, Mickaël Cargnel, Virginie Roupie, Fabienne Rauw, Bénédicte Lambrecht

**Affiliations:** Sciensano, Brussels, Belgium (M. Steensels, S. Van Borm, T. Van Den Berg, M. Cargnel, V. Roupie, F. Rauw, B. Lambrecht);; Animal Health Vlaanderen, Torhout, Belgium (P. Gelaude)

**Keywords:** Low pathogenicity avian influenza, highly pathogenic avian influenza, outbreak, non-notifiable, influenza, H3N1, viruses, Belgium, respiratory infections, poultry

## Abstract

In 2019, an outbreak of avian influenza (H3N1) virus infection occurred among commercial poultry in Belgium. Full-genome phylogenetic analysis indicated a wild bird origin rather than recent circulation among poultry. Although classified as a nonnotifiable avian influenza virus, it was associated with reproductive tropism and substantial mortality in the field.

Avian influenza is a highly contagious viral disease of poultry, able to infect all species of birds (*1*). Of the 2 existing pathotypes, low pathogenicity avian influenza (LPAI) and highly pathogenic avian influenza (HPAI), infection with LPAI virus is mostly undetected in a flock but can cause some respiratory signs, egg drop, lethargy, and limited mortality (*2**,**3*). Avian influenza H5/H7 subtypes are all notifiable to the authorities in Europe because adaptive mutations can influence the polybasic nature of the hemagglutinin (HA) cleavage site, resulting in HPAI emergence (*4*). For all other avian influenza subtypes, only those with an intravenous pathogenicity index (IVPI) >1.2 must be reported (*5*).

## The Study

**Figure 1:**
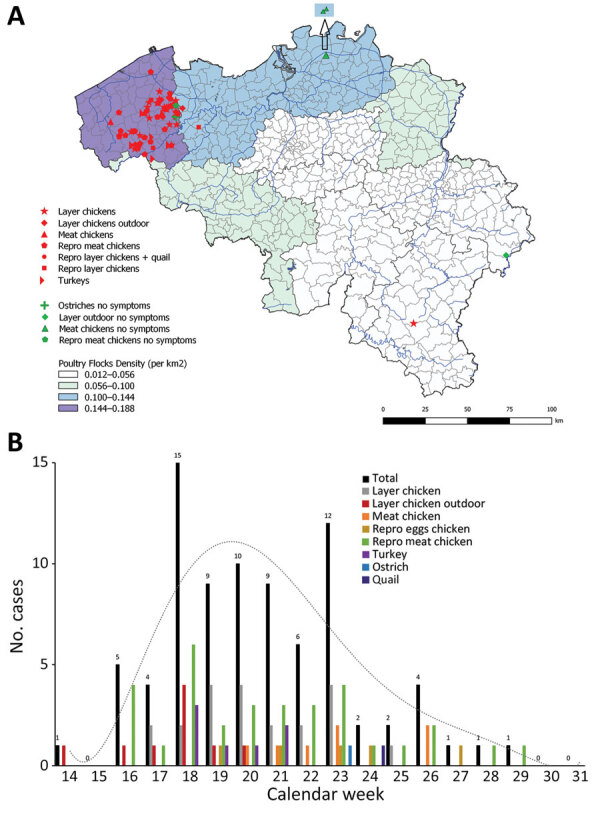
Outbreaks of avian influenza (H3N1) virus among poultry in Belgium, 2019. A) Geographic distribution; B) weekly number of newly identified farms with avian influenza (H3) and poultry species involved. Repro, reproduction.

In January 2019, H3N1 virus was isolated from an outdoor laying hens farm in Belgium. The affected flock was culled, but 3 months later (April), H3N1 virus was again detected on this farm (*5**–**8*), indicative of incomplete virus elimination. Since then, 82 holdings in northwestern Belgium were infected with H3N1 virus in a 16-week period, involving different poultry species and types of farms (Figure 1, panels A, B). In southern Belgium, only 1 farm was clinically affected, for which a direct link with a positive farm in northwestern Belgium was identified. Also, 5 farms with asymptomatic poultry tested positive for H3N1 virus (Figure 1, panel A), without a clear link to the category of farm or species (2 broiler, 1 outdoor laying, 1 ostrich, and 1 broiler breeder farm). In France, 3 farms were H3N1-positive; 2 showed a direct link with a company from the affected area of Belgium (*9*).

The clinical picture in the field started mostly with discolored eggs for laying hens and breeders, followed by an increased mortality rate of up to 60% in breeder hens and 40% in laying hens. In addition, a marked egg drop, up to 100%, was reported. Recovered animals never fully regained their laying potential. A second wave of mortality caused by secondary infection was detected later. During autopsy, specific lesions were detected (e.g., point bleeding in the proventriculus, trachea, and brain; congestion of the kidneys, ovary, and brain; and atrophy of the oviduct, resulting in egg yolk leakage and peritonitis).

For the virus strain isolated in April from poultry on the index farm (A/Gallus gallus/Belgium/3497_0001/2019), hereafter called the 3497_April isolate, the IVPI was 0.13 (Sciensano Bioethics Committee approval 20170117-01), indicating a low pathogenicity phenotype. The complete coding sequences of the HA and neuraminidase (NA) genes, identified by Sanger sequencing, of the strains isolated in January (A/Gallus gallus/Belgium/609/2019 [H3N1]; GenBank accession nos. MK972679 [H3]; MK972680 [N1]) and April (A/Gallus gallus/Belgium/3497_0001/2019 [H3N1]; GenBank accession nos. MK972681 [H3] and MK972682 [N1]) confirmed their relationship. These results demonstrated that the virus from January 2019 had evolved into a poultry-adapted strain by April 2019, with a deletion in the stalk of the NA gene, previously described as crucial for adaptation from wild birds to poultry (*10**,**11*). Next-generation sequencing of the 3497_April isolate demonstrated that the most closely related publicly available sequence of all 8 complete genome segments (accession nos. MN006980–7) linked to avian influenza viruses from Eurasia that were circulating in wild birds with occasional spillover to poultry (Table 1). Using neighbor-joining and maximum-likelihood phylogenetic analyses, we were unable to clearly associate the 2019 outbreak virus with recent virus isolates from poultry (data not shown).

**Table 1:** Most homologous publicly available sequence for each gene segment of A/Gallus gallus/Belgium/3497_0001/2019 (H3N1) virus*

Gene	**Closest BLAST hit (closest poultry BLAST hits)**		
	Sequences	Accession no.	Nucleotide identity, %
PB2	A/tufted duck/Georgia/1/2012 (H2N3) (A/chicken/Hubei/ZYSJF15/2016[H9N2])	MF147767.1 (KY415880.1)	97.74 (96.66)
PB1	A/northern shoveler/Egypt/MB-D-695C/2016 (H7N3) (A/chicken/Sichuan/k141/2017[H5N6])	MN208053.1 (MH715337.1)	98.58 (98.24)
PA	A/mallard duck/Netherlands/56/2015 (H3N2) (A/pigeon/Anhui/08/2013[H3N8])	MF755261.1 (KJ579961.1)	98.68 (96.68)
HA	A/Mallard/Netherlands/37/2015 (H3N8) (A/chicken/Viet Nam/HN-1724/2014[mixed])	MK414733.1 (MK963734.1)	98.32 (96.68)
NP	A/duck/Mongolia/543/2015 (H4N6) (A/chicken/Italy/22A/1998[H5N9], A/chicken/Vietnam/HU1-976/2014[H9N2])	LC121413.1 (CY022624.1, LC069933.1)	98.50 (96.79, 96.64)
NA	A/mallard duck/Georgia/7/2015 (H6N1) (A/chicken/Vietnam/HU4-26/2015[H6N1], A/chicken/France/150169a/2015[H5N1])	MF694086.1 (LC339717.1, KU310449.1)	97.94 (96.78, 96.45)
M	A/mallard/Netherlands/89/2017 (H4N6) (A/domestic duck/Georgia/9/2016[H4N6])	MK192396.1 (MF694025.1)	98.91 (98.11)
NS	A/mallard duck/Netherlands/31/2013 (H10N7) (A/chicken/France/150169a/2015[H5N1], A/chicken/Korea/C47/2009(H9N2))	KX979173.1 (KU310451.1, KY785842.1)	99.19 (98.04, 97.46)

*Determined by BLASTn analysis (https://blast.ncbi.nlm.nih.gov/Blast.cgi). NA, neuraminidase; M, matrix; neuraminidase; HA, hemagglutinin; matrix; NS, nonstructural; PA, polymerase acidic; PB, polymerase basic.

We performed in vivo infection studies (Sciensano Bioethics Committee approval 20180222-01) with the 3497_April isolate, obtained after inoculation of a lung/trachea homogenate into 9-day-old embryonated specific pathogen–free chicken eggs. First, we inoculated 11-week-old specific pathogen–free White Leghorn chickens oculonasally with 10^6^ 50% egg infectious dose (EID_50_) of the 3497_April virus, but no clinical signs were noted over 14 days. At the end of the experiment, 100% (by nucleoprotein [NP] competition ELISA; IDVet, https://www.id-vet.com) and 90% (by standard hemagglutination inhibition [HI]) of the infected birds had seroconverted, confirming infection. Subsequently, to evaluate a possible synergistic effect of a potential bacterial cofactor, we inoculated a non–antibiotic treated lung/trachea homogenate from an H3N1 virus–confirmed animal simultaneously with the H3N1 isolate. At 6–11 days postinoculation (dpi), 40% of the birds exhibited clinical signs (lethargy, clouded eyes, and ruffled feathers) and some died (10% on 13 dpi). This co-infection did not reproduce the clinical picture observed in the field, indicating that a bacterial cofactor would not be necessary to produce the clinical signs seen in the field.

**Figure 2:**
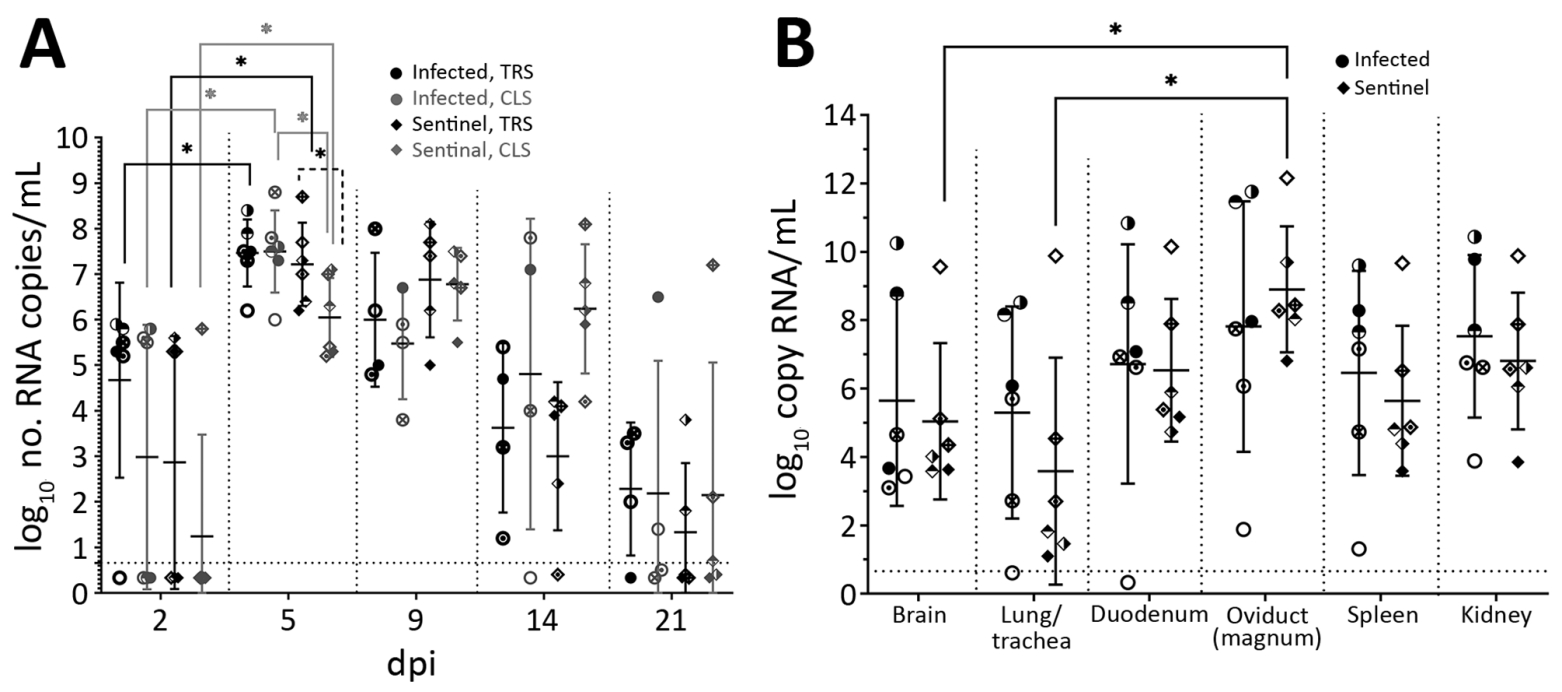
Viral presence in birds during experimental infection with avian influenza (H3N1) isolated from poultry in Belgium, 2019. The individual excretion values are shown (different patterns in circles and diamonds correspond to individual birds) in addition to the average group value ± SDs (error bars). Asterisks (*) indicate statistically relevant differences between time points, groups, or organs, p<0.05. Note: for diluted viral isolates the correspondence between log10 (no. copies/mL) and log10 (50% egg infectious dose/mL) is a difference of ≈1 log less in egg infectious dose. The exact link (by determining the titer of infectious virus in the samples) was considered beyond the scope of this article. A) Viral RNA excretion of infected and sentinel birds at different points after infection, by respiratory gastrointestinal tract samples. B) Viral presence in the organs of infected and sentinel birds at time of death or the end of the experiment (21 dpi), determined by real-time reverse transcription PCR. Viral RNA excretion or presence is expressed as the logarithm of the number of viral RNA copies/mL as quantification was performed relative to an external curve during real-time reverse transcription PCR analysis. CLS, cloacal samples; dpi, days postinfection; repro, reproduction; TRS, oropharyngeal swab samples.

Because the clinical effect in the field was greater for layer hens in production, we performed a third in vivo evaluation in 24-week-old conventional Bovans Brown laying hens, avian influenza negative serologically (NP ELISA and HI [*5*]) and virologically (real-time reverse transcription PCR [*7*]) at arrival. Six chickens were infected with 10^6^ EID_50_/bird and 6 noninfected sentinel birds were added at 1 dpi. Of the 6 infected birds, 2 died at 6 and 7 dpi, showing either no or mild clinical signs at 5 dpi. Autopsy of each bird that died confirmed some of the lesions described for birds in the field (pronounced congestion in the brain, kidney, and intestine; and kidney enlargement). Among the sentinel birds, 1 bird died at 8 dpi without prior clinical signs or clear pathologic lesions during autopsy but demonstrated a higher amount of viral RNA (10^5^) in the organs compared with the other sentinel birds at the end of the study (21 dpi) (Figure 2, panel B). 

Analysis of viral RNA excretion (Figure 2, panel A) demonstrated viral shedding from all infected and sentinel birds and a significant increase (p*<*0.05) from 2 dpi to 5 dpi in both groups (nonparametric Wilcoxon signed-rank test). Excretion did not differ between sentinel and infected chicken except at 5 dpi, when cloacal excretion by sentinel chickens was significantly higher (p<0.05) than that by infected chickens (nonparametric Mann-Whitney test). No significant difference was observed between oropharyngeal and cloacal excretions, except at 5 dpi oropharyngeal excretion by sentinel chickens was higher (p<0.05) (nonparametric Wilcoxon signed-rank test). 

## Conclusions

The viral RNA excretion period of the H3N1 virus under investigation demonstrates an extended period of virus excretion compared with that generally expected for LPAI viruses. LPAI virus excretion mostly starts 1–2 days after infection and continues for 7–10 days, peaking on day 5, then quickly declining because of immunity onset (*12**,**13*). In sentinel birds, the viral RNA concentration was significantly higher (p<0.05) in the oviduct than in the brain and lung/trachea (nonparametric Friedman test) (Figure 2, panel B); differences with other organs were not significant. In infected chickens, this trend was confirmed, albeit not significantly. The systemic distribution, although limited to chickens during laying, is a typical feature for HPAI and is not expected for LPAI. All birds had seroconverted at 9 dpi, as measured by NP competition ELISA, and all but 1 infected bird seroconverted at 9 dpi and all surviving sentinels had seroconverted at 14 dpi as measured by HI (Table 2).

**Table 2:** Seroconversion of sentinel birds and birds infected with A/Gallus gallus/Belgium/3497_0001/2019 (H3N1) virus

Bird	9 dpi					14 dpi						21 dpi			
	ELISA-NP†		HI‡			ELISA-NP†			HI‡			ELISA-NP†		HI‡	
	S/N, %	IR	log 2	IR		S/N, %	IR		log 2	IR		S/N, %	IR	log 2	IR
Infected 1	4.3	+	6	+		4.8	+		10	+		5.5	+	8	+
Infected 2	5.0	+	6	+		5.6	+		9	+		4.7	+	9	+
Infected 3	5.8	+	6	+		4.8	+		9	+		4.3	+	9	+
Infected 4	10.2	+	3	–		9.0	+		3	–		12.6	+	3	–
Infected 5§	NA	NA	NA	NA		NA	NA		NA			NA	NA	NA	NA
Infected 6§	NA	NA	NA	NA		NA	NA		NA			NA	NA	NA	NA
Average (SD)	6.3 ±2.6		5.3 ±1.5			6.0 ±2.0			7.8 ±3.2			6.8 ±3.9		7.3 ±2.9	
Sentinel 1	17.6	+	2	–		5.3	+		7	+		4.8	+	9	+
Sentinel 2	5.9	+	2	–		4.9	+		7	+		4.8	+	7	+
Sentinel 3	7.7	+	2	–		4.9	+		6	+		4.6	+	8	+
Sentinel 4§	NA	NA	NA	NA		NA	NA		NA			NA	NA	NA	NA
Sentinel 5	5.7	+	3	–		4.7	+		8	+		4.8	+	9	+
Sentinel 6	5.6	+	2	–		4.4	+		7	+		5.5	+	8	+
Average (SD)	8.5 ±5.1		2.2 ±0.4			4.8 ±0.3		7.0 ±0.7				4.9 ±0.4		8.2 ± 0.8	

*dpi, days postinfection; ELISA-NP, nucleoprotein competition ELISA; HI, hemagglutination inhibition; IR, immune response; NA, not applicable because of bird death; S/N, signal-to-noise ratio; +, positive immune response; –, negative immune response.  †(IDVet, https://www.id-vet.com/). ELISA data are presented as the percentage of competition detected, the S/N. ‡HI test with the homologous H3N1 antigen. HI data are presented as log2 of the HI titer.

No virus transmission to humans was reported. All 20 analyzed conjunctival swab specimens from farmers without clear influenza-like symptoms on the infected farms were negative for H3N1 virus by the Belgian National Reference Center of Influenza (I. Thomas, Sciensano, pers. comm., 2019 Jul 31). 

Although this virus was classified as LPAI according to official definitions using IVPI or molecular criteria, it was more pathogenic in laying hens than expected for a non-notifiable LPAI. Increased pathogenicity was shown by systemic distribution, clinical signs, and increased transmissibility in poultry (especially laying hens) under field and experimental conditions.
